# Predictive Value of Neutrophil to Lymphocyte Ratio in the Diagnosis of Significant Prostate Cancer at Initial Biopsy: A Comparison with Free Percent Prostate Specific Antigen, Prostate Specific Antigen Density and Primary Circulating Prostate Cells

**DOI:** 10.31557/APJCP.2019.20.11.3385

**Published:** 2019

**Authors:** Nigel P Murray, Cynthia Fuentealba, Eduardo Reyes, Marco Antonio López, Aníbal Aníbal, Simona Minzer, Lorena Munoz, Shenda Orrego, Eghon Guzman, Lucas Arzeno

**Affiliations:** 1 *Consultant in Hematology, Department of Medicine, *; 3 *Consultant Urologist, Department of Urology, *; 6 *Physican General Medicine, *; 7 *Consultant Internal Medicine, Department of Medicine, Hospital de Carabineros de Chile, Simón Bolívar, Ñuñoa,*; 2 *Professor Hematology and Head CTC Unit, Faculty of Medicine, University Finis Terrae, Av Pedro de Valdivia, Providencia, *; 4 *Consultant Urologist, Department of Urology, Hospital DIPRECA, Vital Apoquindo 1200, Las Condes, *; 5 *Faculty of Medicine, University Diego Portales, Manuel Rodríguez Sur, *; 8 *Tutor, Faculty of Medicine, University Mayor, San Pio X 2245, Providencia, Santiago, Chile. *

**Keywords:** prostate cancer, neutrohil/lymphocyte ratio, circulating prostate cells

## Abstract

**Introduction:**

An elevated serum PSA is the only biomarker routinely used in screening for prostate cancer to indicate a prostate biopsy. However, it is not specific for prostate cancer and the neutrophil/lymphocyte ratio has been suggested as an alternative. We present a prospective study of men with an elevated PSA and compare the neutrophil/lymphocyte ratio, free percent PSA, PSA density and the presence of circulating prostate cells to detect clinically significant prostate cancer at first biopsy.

**Patients and Methods:**

Prospective study of consecutive men with a PSA 4-10 ng/ml referred for initial prostate biopsy, the results were compared with the neutrophil/lymphocyte ratio, free percent PSA and PSA density. Circulating prostate cells (CPCs) were detected using immunocytochemistry. The blood sample was taken immediately before the prostate biopsy.

**Results:**

1,223 men participated, 38% (467) of whom had prostate cancer detected, of these 322 were clinically significant. The area under the curves were for neutrophil/lymphocyte ratio, free percent PSA, PSA density and CPC detection were 0.570, 0.785, 0,620 and 0.844 respectively. Sensitivity/specificity were 0.388/0.685, 0.419/0.897, 0.598/0.624 and 0.966/0.786 respectively. The neutrophil/lymphocyte ratio did not differentiate between benign and malignant disease.

**Conclusions:**

The neutrophil/lymphocyte ratio did not discriminate between benign and malignant prostatic disease in patients with a PSA between 4-10ng/ml.

## Introduction

Serum prostate specific antigen (PSA) is the only biomarker routinely used for the early detection of prostate cancer (PC), but it is not a perfect test. Although PSA is highly specific for prostate, an elevated level is not specific for cancer, being increased in benign pathologies (Pungalia et al., 2006). Consequently, approximately 70% of men with an increased serum PSA between 4 and 10 ng/ml do not have prostate cancer (Bray et al., 2018) and thus undergo unnecessary prostate biopsies (PB). Furthermore, of the prostate cancers detected, it has been reported that up to 50% do not require treatment (Draisma et al., 2009). The use of free percent PSA, PSA density have been used to try to compensate for the low specificity of total PSA but their use remains controversial (Nordstrom et al., 2017). The need to identify biomarkers that predict the presence of clinically significant prostate cancer in this group of men with an increased PSA and thus decrease the number of “un-necessary” prostate biopsies.

Systemic inflammation plays a role in the development and progression of prostate cancer (Nuhn et al., 2014; Langen et al., 2015) and could be a possible marker for the detection of prostate cancer (Kawahara et al., 2015; Maeda et al., 2016; Oh et al., 2016). One marker of such inflammation is the neutrophil to lymphocyte ratio (NLR) which combines the circulating neutrophil and lymphocyte counts. The NLR has been shown to be higher in cancer patients than those with benign hyperplasia but did not differentiate cancer patients from those with prostatitis (Gokce et al., 2015). Similarly in a study of 168 men the N/L ratio failed to differentiate between malignant and benign pathologies (Khosropanah et al., 2018).

However many of the reported studies excluded men with “medically active illness” (Oh et al., 2016), those using anti-inflammatory drugs, inflammatory disease such as lupus, prostatitis (Kawahara et al., 2015; Oh et al., 2016) and as such may not represent a typical screening population. 

One reported biomarker is the detection of primary circulating prostate cells, in men with prostate cancer there is, at least, one subpopulation of cancer cells that disseminate early, firstly to the neurovascular structures and then to the circulation (Moreno et al., 1992). The number of these cells is very small; however, these mCPC can be detected using immunocytochemistry with a combination of anti-P504S (methyl-acyl-CoA racemase) and anti-PSA monoclonal antibodies. The use of the biomarker P504S, although not prostate specific (Zhou et al., 2002), has facilitated the differentiation between normal, dysplastic and malignant tissues in prostate biopsy samples. Normal or benign cells do not express P504S, whereas cells arising from prostatic intraepithelial neoplasia (PIN) or cancer are positive (Beach et al., 2002). The release of P504S negative CPCs has been reported in benign prostatic disease Murray et al., 2013) and that of circulating epithelial cells in inflammatory bowel disease (Pantel et al., 2012). Thus, the NLR may be association with the detection of CPCs, benign or malignant. The sequential use of CPC detection in men with a raised total PSA has shown a high negative predictive value (Murray et al., 2016), and may reduce the number of men undergoing prostate biopsy without missing a significant number of clinically important prostate cancer (Murray et al., 2017).

We present a study of consecutive men who underwent initial prostate biopsy for an increased total PSA, and the diagnostic yield of free percent PSA, PSA density, the NLR ratio and the presence of primary CPCs.

## Materials and Methods

We prospectively studied all men undergoing an initial trans-rectal ultrasound guided (TRUS) prostate biopsy at the Hospital Carabineros of Chile between January 2009 and May 2015. Indications for a TRUS biopsy were an elevated total PSA, defined as > 4.0 ng/mL, or a digital rectal examination (DRE) abnormal or suspicious of cancer, defined as the presence of a nodule, areas of indurations, or asymmetry in the size of the lateral lobes (Campbell et al., 2011). 

Prior to the biopsy and after written, informed consent the following data and blood samples were taken:

a) age

b) serum PSA (ng/ml) taken before the DRE and pre-biopsy using the Siemens Advia CentaurXR^®^ assay;

c) serum percent free PSA, taken at the same time as the serum PSA using the Siemens Advia CentaurXR^® ^assay.

d) full blood count using the Beckton-Coulter H250^®^ to determine the absolute neutrophil and lymphocyte counts, the ratio was obtained using the absolute neutrophil count divided by the absolute lymphocyte count.

e) prostatic volume: Trans rectal ultrasonography of the prostate was performed

using an endocavitary convex probe with a 6.5MHz transducer (Hitachi, model EVP-V33). Measures of the triaxial distances of the prostate were taken in its larger diameter and the total volume was calculated by the following formula: volume = 0.52 x transverse diameter x antero-posterior diameter x longitudinal diameter.

f) TRUS biopsy: all biopsies were standard 12 core, performed trans-rectally under ultrasound guidance by an experienced urologist using an 18 gauge Tru-Cut needle. Each core was sampled separately, stored in formaldehyde and sent for pathological assessment. A biopsy was defined as positive only when adenocarcinoma as observed in the final histological evaluation. In positive samples, the Gleason score, number of positive cores and maximum percent infiltrated was recorded. The pathological analysis and reports were performed by a single dedicated uro-pathologist.

*g) Detection of primary circulating prostate cells*

Immediately before the biopsy, an 8mL venous blood sample was taken and collected in a tube containing EDTA (Becton-Dickenson-Vacutainer). Samples were maintained at 4^o^C and processed within 48 hours. The prostate biopsy and CPC detection were independently evaluated with the evaluators being blinded to the clinical details and results of the biopsy or CPC test.

*Collection of CPCs*

Mononuclear cells were obtained by differential centrifugation using Histopaque 1,077 (Sigma-Aldrich), washed, and suspended in a 100 µL aliquot of autologous plasma. 25 µL aliquots were used to make slides (silanized, DAKO, USA), were dried in air for 24 hours and fixed in a solution of 70% ethanol, 5% formaldehyde, and 25% phosphate buffered saline (PBS) pH 7.4 for five minutes and finally washed three times in PBS pH 7.4 .

*Immunocytochemistry*

CPCs were detected using a monoclonal antibody directed against PSA, clone 28A4 (Novo Castro Laboratory, UK), and identified using an alkaline phosphatase-anti alkaline phosphatase based system (LSAB2, DAKO, USA), with new fuchsin as the chromogen. Positive samples underwent a second process with anti-P504S clone 13H4 (DAKO, USA) and were identified with a peroxidase based system (LSAB2, DAKO, USA) with DAB (3, 3 diaminobenzidine tetrahydrochloride) as the chromogen. A CPC was defined according to the criteria of ISHAGE (International Society of Hemotherapy and Genetic Engineering) (Borgen et al., 1999) and the expression of P504S according to the Consensus of the American Association of Pathologists (Rubin et al., 2002). A CPC was defined as a cell that expressed PSA and P504S. 

Slides were analyzed manually, stained cells were photographed using a digital camera and from the digital images determined if CPCs were present or absent and the total number of CPCs detected by one trained observer. A test was considered positive when at least 1 cell/8mL of blood was detected. 

*Analysis of the Results*

The discrimination of the diagnostic tests was defined using the normal parameters: sensitivity, specificity, true positive (TP); false positive (FP), false negative (FN), and true negative (TN). The predictive values, positive (PPV) as well as negative (NPV), were evaluated and the areas under the curve calculated and compared. The potential number of biopsies avoided for each method was calculated and the Gleason scores of missed cancers recorded. 

In addition, using the criteria of Epstein et al., (1994), the number of cancers needing active treatment and active observation were registered for each test, whether the test was positive or negative, in order to determine the clinical significance of each test used.

*Statistical Analysis*

Descriptive statistics were used for demographic variables, expressed as mean and standard deviation in the case of continuous variables with a normal distribution. In case of an asymmetrical distribution, the median and interquartile range (IQR) values were used. Noncontiguous variables were presented as frequencies. The Shapiro-Wilk test was used to determine a normal distribution. The Student *t*-Test was used to compare continuous variables with a normal distribution, the Mann-Whitney test for ordinate and continuous variables with a non-normal distribution, and the Chi-squared test for the differences in frequency. The diagnostic yield for the four tests were analyzed using standard parameters. For this purpose, patients were classified as having or not having prostate cancer. Statistical significance was defined as a value less than 0.05; all tests were two-sided. Area under the curve analysis was performed using the online program Vassarcalc. The sample size was calculated using the on-line MedCalc, assuming 40% of biopsies would be positive for cancer of which 25% would be clinically insignificant in a Chilean population with a PSA of 4-10 ng/ml and using an alpha value of 0.05, a power to detect of 80% with a confidence interval of 95% then a minimum of 843 patients would need to be included in the study.

*Ethical Considerations*

The study was approved by the hospital ethics committee and performed in accordance with the Declaration of Helsinki.

**Table 1:** Patient Characteristics According to Biopsy Results

	No cancer (N=756)	Cancer (N=467)	P-value
Mean age ± SD (years)	64.2 ± 9.1	65.5 ± 9.5	< 0.001
PSA (ng/ml) median (IQR)	5.51 (4.40-7.51)	5.90 (4.80-9.12)	< 0.001
percentage free PSA median (IQR)	18 (14-24)	11 (9-14)	< 0.001
PSA density median (IQR)	0.13 (0.09-0.17)	0.16 (0.11-0.25)	< 0.001
N/L ratio median (IQR)	2.00 (1.53-2.79)	2.21 (1.48-3.33)	p=0.71
CPC (+)	143/756 (18.9%)	407/467 (87.2%)	< 0.001

CPC, circulating prostate cell; N/L ratio, neutrophil to lymphocyte ratio; IQR, inter-quartile range

**Table 2:** Detection of Clinically Significant Prostate Cancer According to Test

Test negative	N=	Cancer needing treatment	Percentage of significant cancer not detected	Test positive	N=	Cancer needing treatment	Percentage of benign biopsies
CPC (-)	696 (57%)	12	12/353 (3%)	CPC (+)	527 (43%)	341/353 (97%)	186/527 (35%)
% free PSA >10%	985 (80%)	205	205/353 (58%)	% free PSA < 10%	238 (20%)	148/353 (42%)	90/238 (38%)
PSA density < 0.165	685 (56%)	142	142/353 (40%)	PSA density >0.15	538 (44%)	211/353 (60%)	327/538 (61%)
N/L ratio < 2.5	812 (66%)	216	216/353 (61%)	N/L ratio > 2.5	411 (34%)	137/353 (34%)	274/411-67%

CPC, circulating prostate cell; N/L, neutrophil to lymphocyte

**Table 3:** Sensitivity, Specificity and Positive and Negative Predictive Values for Each Test

	sensitivity	specificity	PPV	NPV	PLR	NLR
N/L ratio	0.388	0.685	0.333	0.734	1.23	0.36
	(95% CI 0.337-0.441)	(95% CI 0.652-0.716)	(95% CI 0.288-0.382)	(95% CI 0.702-0.764)	(95% CI 1.05-1.45)	(95% CI 0.32-0.41)
percentage free PSA	0.419	0.897	0.622	0.792	4.05	0.26
	(95% CI 0,368-0.473)	(% CI 0.874-0.916)	(95% CI 0.556-0.683)	(95% CI 0.765-0.817)	(95% CI 3.22-5.11)	(95% CI 0.23-0.30)
PSA density	0.598	0.624	0.392	0.793	1.59	0.26
	(95% CI 0.544-0.649)	(95% CI 0.591-0.656	(95% CI 0.351-0.435)	(95% CI 0.760-0.822)	(95% CI 1.41-1.80)	(95% CI 0.23-0.30)
CPC	0.966	0.786	0.647	0.983	4.52	0.02
	(95% CI 0.940-0.982)	(95% CI 0.757-0.813)	(95% CI 0.604-0.688)	(95% CI 0.969-0.991)	(95% CI 3.97-5.14)	(95% CI 0.01-0.03)

PPV, positive predictive value; NPV, negative predictive value; PLR, positive likelihood ratio; NLR, negative likelihood ratio; N/L, neutrophil to lymphocyte ratio; CPC, circulating prostate cell

## Results

1223 men participated in the study, 467/1,223 (38.2%) had a biopsy positive for prostate cancer of which 114/467 (24.4%) complied with the Epstein criteria for active observation. 296/467 (63.2%) were Gleason 65 tumors, 145/467 (31.0%) Gleason 7 and 27/467 (5.8%) were Gleason 8 or higher. Table 1 shows the data base variables according to the biopsy results.

Patients with a biopsy positive for cancer were significantly older, had a higher median PSA, a lower % free PSA, a higher PSA density and were positive for circulating prostate cells, there was no significant difference between the N/L ratios.

The median N/L ratio was not significantly different between men CPC positive and CPC negative (2.21 (IQR 1.67-3.15) versus 1.96 (IQR 1.46-2.80) P=0.52); between men with a PSA density <0.15 versus > 0.15 (2.03 (IQR 1.53-2.76) versus 2.23 (IQR 1.45-3.39) P=0.24) or percent free PSA <10% versus > 10% (2.24 (IQR 1.54-3.53) versus 2.00 (IQR 1.47-2.85) P=0.16).

The area under the curves were CPC 0.844, free percent PSA 0.785, PSA density 0.620 and N/L ratio 0.570 respectively. The predictive accuracy, showed that CPC detection was significantly better than percent free PSA, PSA density and N/L ratio (P<0.001); percent free PSA was significantly better than PSA density and N/L ratio (P<0.001) and that the PSA density was superior to the N/L ratio (P<0.05).

*Detection of clinically significant prostate cancer*

Using the cutoff values of: CPC positive, percent free PSA ≤ 10%, PSA density ≥0.15 and N/L ratio >2.5 to determine the need for a prostate biopsy to detect clinically significant prostate cancer the results are shown in Table 2. For the purpose of the analysis, we used the defined cutoff values to determine the number of biopsies that potentially could be avoided and the number of clinically significant prostate cancers that would be missed.

The number of prostate biopsies that potentially could be avoided was significantly higher using the percent free PSA, followed by the N/L ratio, there was no significant difference between PSA density and CPC detection. However, the cost of this reduced number of biopsies was the number of clinically significant prostate cancers that would be missed. Both percent free PSA and the N/L ratio using the established cutoff values failed to detect approximately 60% of clinically significant prostate cancers. The PSA density cutoff value of >0.15 failed to detect 40% of clinically significant prostate cancers, while that of CPC failed to detect 3%.

Table 3 shows the specificity, sensitivity, positive and negative predictive values and likelihood ratios for each test.

## Discussion

The aim of this study was to evaluate the diagnostic yield of the neutrophil/lymphocyte ratio with respect to the prostate biopsy results in a typical screening population. Published results have been contradictory, Gokce et al., (2015) reported that the ratio was higher in men with prostate cancer and with higher Gleason scores; however it did not differentiate between men with prostate cancer or prostatitis. The authors concluded that inflammation had a role in the development of prostate cancer. Kawahara et al., (2015) in a retrospective study reported that the higher the N/L ratio the higher the incidence of prostate cancer as did Adhyatma et al., (2019), while that of Kamali et al., (2018) did not find an association. Our prospective study did not find an associated with clinically significant prostate cancer, and the predictive value was significantly inferior to that of PSA density, free percent PSA and primary CPCs. Nor was there an association between the N/L ratio and the presence or absence of CPCs. In benign inflammatory conditions, P504S negative CPCs have been detected (Murray et al., 2013), as well as EpCAM positive cells in patients with inflammatory bowel disease (Pantel et al., 2012). It is possible that local inflammation is not sufficient to alter the N/L ratio. The study by Kawahara et al., (2015) reported an area under the curve of 0.58 for the N/L ratio and the presence of prostate cancer, which is similar to our reported value of 0.57. However, in their patient population they reported an association with a positive biopsy. Their study excluded 422 patients from the initial population of 4,335 men; we did not exclude patients from our screening population. A possible explanation of the differing results is that in older men there is a “general inflammatory state” that masks any elevation of the N/L ratio caused by prostate cancer. Thus, the N/L ratio does not discriminate between benign histology (hyperplasia and prostatitis) and prostate cancer (Gokce et al., 2015). A second alternate is that the frequency of prostatitis in the general population may be sufficient to mask any differences between benign and malignant prostate disease. There is no definite evidence that chronic prostatitis is associated with systemic inflammation (Sutcliffe et al., 2007) or the elevation of a systemic inflammatory marker in chronic prostatitis and prostate cancer (Sfanos et al., 2012).

The use of the N/L ratio with a cutoff value of 2.5 did not differentiate between patients with clinically significant prostate cancer and those patients who did not need treatment (combined benign pathology and non-significant cancer). A “negative test” included 61% of all clinically significant prostate cancers and was similar to that of free percent PSA with a cutoff value of 10%. Of patients with an elevated N/L ratio 57% of these biopsies did not detect a clinically significant prostate cancer. This finding is in keeping with the results of Khosropanah et al., (2018). However it contrasts with the findings that the N/L ratio was positively associated with the Gleason score (Gokce et al., 2015), and related to unfavorable clinicopathological outcomes (Lee et al., 2016).

None of the four tests had an excellent positive predictive value, however, CPC negative patients had a negative predictive value of over 98%, or in other words, these patients could avoid undergoing prostate biopsy. For the other three methods, the negative predictive values were similar.

In conclusions,the ideal biomarker for the detection of prostate cancer in a screening population is one that detects clinically significant cancers, does not detect indolent cancer and has a high negative predictive value to avoid unnecessary biopsies. The use of the N/L ratio did not fulfill these requirements and did not discriminate between patients with clinically significant prostate cancer, indolent cancer and benign prostatic disease in a Chilean screening population with a PSA of 4-10 ng/ml. As such, the use of the N/L ratio in predicting prostate cancer prior to prostate biopsy is not supported by these findings.
